# Longitudinal Changes in the Endothelial Activation and Stress Index (EASIX) in Patients with Preeclampsia

**DOI:** 10.3390/diagnostics16132007

**Published:** 2026-06-27

**Authors:** Anna Sophie Scholz, Annabel Kussner, Michael Elsässer, Lara Meike Tretschock, Julia Spratte, Thomas Luft, Cahit Birdir, Stephanie Wallwiener, Alexandra von Au

**Affiliations:** 1 Department of Gynecology and Obstetrics, Heidelberg University Hospital, 69120 Heidelberg, Germany; 2 Department Medicine V, Hematology, Oncology, Rheumatology, Heidelberg University Hospital, 69120 Heidelberg, Germany; 3 Department of Obstetrics and Fetal Medicine, University Hospital Halle (Saale), 06120 Halle, Germany

**Keywords:** preeclampsia, endothelial dysfunction, EASIX, adverse outcome

## Abstract

**Background:** Endothelial dysfunction is a central pathophysiological hallmark of preeclampsia. Laboratory and clinical features of preeclampsia can rapidly deteriorate and evidence on appropriate surveillance strategies is scarce. We aimed to evaluate the prognostic value of longitudinal changes in the “Endothelial Activation and Stress Index” (EASIX) for adverse outcomes in patients with preeclampsia. **Methods:** Patients with preeclampsia who delivered at Heidelberg University Hospital between 2017 and 2022 were included in this retrospective analysis. We assessed EASIX, derived from lactate dehydrogenase, creatinine and platelets, longitudinally between first admission and diagnosis of an adverse outcome. Composite adverse outcomes included pulmonary edema, HELLP syndrome, kidney injury, eclampsia, postpartum hemorrhage and death. We applied logistic and mixed linear regression modeling adjusted for age, gestational age and body mass index. **Results:** In total, 1733 EASIX measurements of 443 patients were included in the analysis, of which 81 patients experienced an adverse outcome. Both the first EASIX (aOR 2.81 [1.86;4.37]) and the absolute change per day (aOR 4.35 [1.77; 12.05]) were independently associated with adverse outcomes. Addition of the absolute change in EASIX to the model including the first EASIX (AUC 0.74 [0.68–0.81]) did not substantially improve the discriminatory performance (AUC 0.76 [0.70; 0.82]). Linear mixed regression modeling demonstrated that patients with adverse outcomes had a steeper rise in EASIX compared to patients without adverse outcomes (β = 0.024 [0.013, 0.034]). **Conclusions:** In patients with preeclampsia, EASIX diverged over time with steeper slopes in patients who developed adverse maternal outcomes. Our findings suggest that longitudinal EASIX monitoring may correlate with endothelial dysfunction and capture individual disease dynamics that are not apparent from a single measurement.

## 1. Introduction

Preeclampsia affects up to 8% of all pregnancies [1], thus representing the leading cause for maternal and perinatal morbidity, and is responsible for over 70,000 maternal deaths and 500,000 fetal deaths worldwide per year [2,3]. Delivery may resolve most symptoms of preeclampsia; however, women who experienced preeclampsia will face an increased risk for long-term cardiovascular complications [4,5]. Preeclampsia constitutes one of the most challenging complications in obstetrics as the clinical presentation can be highly heterogeneous and may rapidly progress into severe maternal complications [3]. The management of preeclampsia is challenging as the risk for maternal adverse outcomes needs to be balanced with the risk of prematurity and associated perinatal morbidity.

The Endothelial Activation and Stress Index (EASIX) uniquely integrates markers that reflect different mechanisms of systemic endothelial dysfunction. In view of the highly heterogeneous response pattern of the endothelium, one universal marker often fails to capture the systemic effect of endothelial activation and dysfunction. EASIX combines the typical increased lactate dehydrogenase (LDH) levels due to cell activation, the interaction of endothelial cells with circulating platelets leading to platelet consumption [6], and the affected kidney function mirroring the glomerular endotheliosis (creatinine). EASIX has emerged as a validated marker of mortality in disorders associated with systemic endothelial dysfunction, demonstrating prognostic value for adverse outcomes in various clinical settings [7,8,9,10,11,12,13]. EASIX was originally developed to predict endothelial-related complications after allogenic stem cell transplantation (alloSCT) [14]. The endothelial nature of the marker was recently underlined by a prospective study revealing a correlation of EASIX with glycocalyx thickness, digital perfusion and vascular permeability, and platelet aggregation [15].

Recently, EASIX was introduced in the context of preeclampsia, demonstrating a significant association with preeclampsia-related fetomaternal adverse outcomes [16]. However, the clinical value and prognostic information of longitudinal changes in EASIX beyond a single baseline measurement at admission remains to be explored. Repeated biomarker assessment may offer the opportunity to characterize individual patient dynamics.

Therefore, we aimed to evaluate the additive value of longitudinal changes in EASIX in patients with preeclampsia compared to a single EASIX measurement.

## 2. Materials and Methods

### 2.1. Study Design and Population

This study was a single-center, retrospective analysis based on routinely collected data from the Department of Gynecology and Obstetrics, University Hospital Heidelberg, Heidelberg, Germany. The local ethics committee of the medical faculty of the Heidelberg University (S-680/2023, 4 December 2023) approved the study. All patients who delivered at our department between 2017 and 2022 and had one of the preeclampsia-related ICD-10 (International Classification of Diseases) codes O10, O11, O13, or O14 were eligible for the analysis. Patient records were thoroughly reviewed to confirm the diagnosis of preeclampsia according to the International Society for the Study of Hypertension in Pregnancy (ISSHP) criteria of 2021 which define preeclampsia as elevated blood pressure in pregnancy in combination with any end-organ dysfunction [17].

We excluded patients with diseases that could possibly influence the EASIX value such as severe renal or cardiovascular disease or SARS-CoV-2 infection at any time during pregnancy. In case of more than one pregnancy during the observational period, only the first available pregnancy complicated by preeclampsia was used for the analysis. Further exclusion criteria were any fetal chromosomal, genetic, or structural anomalies and missing data. Diagnosis of any adverse maternal outcome at the time of the first EASIX measurement was an exclusion criterion.

### 2.2. EASIX (Endothelial Activation and Stress Index)

Routine laboratory measurements including the necessary parameters for calculation of EASIX were performed regularly after first assessment until delivery, and the frequency of blood samples depended on the current clinical status of the patient and the clinicians’ medical evaluation. Each patient contributed at least two samples. The number of EASIX assessments was not fixed ranging from 2 to 15 assessments with a median of 3 assessments. EASIX was calculated by lactate dehydrogenase (U/L) × creatinine (mg/dL) divided by platelet count (10^9^ cells per L).

### 2.3. Outcome Definition

The primary outcome definition was based on the preeclampsia core outcome set [18,19] and included the occurrence of any adverse maternal outcome defined as composite of death; eclampsia; the Hemolysis, Elevated Liver enzymes, Low Platelets (HELLP) syndrome defined as increased transaminases more than twice the upper reference limit with platelets <100/nL and at least one hemolysis criterion [17]; acute kidney injury according to the Kidney Disease Improving Global Outcomes (KDIGO) definition (increase in creatinine of ≥0.3 mg/dL [≥26.5 μmol/L] within 48 h, an increase of ≥1.5× from baseline within 7 d, or urine volume <0.5 mL/kg/h for 6 h) [20]; pulmonary edema; disseminated intravascular coagulation; and postpartum hemorrhage.

Previously reported analyses by our group demonstrated a stronger and more consistent association of EASIX with adverse maternal than with perinatal outcomes [16], suggesting that EASIX, as a marker of endothelial dysfunction, primarily reflects maternal organ involvement rather than fetoplacental pathology. We therefore focused on adverse maternal outcomes as the primary endpoint, with perinatal outcomes examined in a secondary analysis. Perinatal outcomes were defined as composite of death during the first week after delivery, stillbirth, preeclampsia-related preterm delivery <34 weeks, placental abruption, respiratory distress syndrome, intraventricular hemorrhage, and necrotizing enterocolitis.

### 2.4. Statistical Analysis

Descriptive analysis shows data as categorial variables and continuous as absolute (relative) numbers, or median (interquartile range), where appropriate. Statistical comparisons in cases versus controls were performed using non-parametric methods. *p*-values < 0.05 were considered statistically significant. Spaghetti plots were used to show the individual trajectories of EASIX during the observation period. Logistic regression models included maternal age and gestational age at first EASIX measurement, the time between last and first EASIX measurement and maternal body mass index. These variables were chosen a priori based on clinical relevance. Here, the longitudinal course of EASIX was summarized by “absolute change per day” calculated using the absolute difference between the first and last EASIX measurement divided by the days between these two measurements as described by others before [21,22]. The observation time ended either with delivery or diagnosis of an adverse outcome whatever occurred first. Our analysis only included EASIX values before the diagnosis of an adverse outcome. Crude and adjusted Odds ratios with 95% confidence intervals were calculated. Receiver operating characteristics curve (ROC) analyses were performed to evaluate the discriminatory power of the EASIX parameters for the association with adverse outcomes. To examine the longitudinal trajectory of EASIX in patients with and without adverse outcomes, we additionally fitted a linear mixed-effects model with EASIX as the repeated outcome. The model was specified as EASIX_ij_ = β_0_ + β_1_·Time_j_ + β_2_·Outcome_i_ + β_3_·Age_i_ + β_4_·BMI_i_ + β_5_·Gestational age_i_ + β_6_·(Time × Outcome)_ij_ + u_i_ + ε_ij_. Time_j_ represents the days after admission and initial EASIX measurement. The primary parameters of interest were β_2_ defined as the difference in EASIX between the two groups and β_6_ defined as the difference in EASIX slopes over time between the two groups. The full model including patient-level random intercepts explained 80.7% (conditional R^2^ = 0.807), with a large proportion of this variance attributable to stable between-patient differences in EASIX levels (ICC = 0.779). Residual normality was assessed using the Shapiro–Wilk test, and a sensitivity analysis with log-transformed EASIX was performed to verify robustness of findings. We chose the untransformed model as the primary analysis to preserve clinical interpretability [23], since absolute changes in EASIX on the original scale can be more directly translated into clinically meaningful thresholds.

Statistical analyses were conducted in Prism 9.5.0 (GraphPad Prism Software, Inc. San Diego, CA, USA) and Python 3.10.11 using the following libraries: pandas (v2.3.2) for data handling, NumPy (v1.26.4) for numerical computation, statsmodels (v0.14.6) for fitting the linear mixed-effects model, Matplotlib (v3.10.7) for all figures, and SciPy (v1.12.0) for supplementary statistical tests. Code was developed with the assistance of Claude Sonnet 4.6 (Anthropic, San Francisco, CA, USA, 2025).

## 3. Results

A total of 443 patients with serial EASIX measurements were included (Supplementary Figure S1). EASIX was assessed at up to 39 days after initial measurement, yielding 1733 individual measurements with mean 3.9 EASIX values per patient (3.90 measurements in the outcome group versus 3.91 in the group without adverse outcomes) and mean 1.27 measurements per day. Adverse maternal outcomes were diagnosed in 81 (18.3%) patients, of which 23 had HELLP syndrome, 43 had acute kidney injury, 29 had postpartum hemorrhage, three had pulmonary edema, and one had eclampsia. No death or disseminated intravascular coagulation occurred. Some patients fulfilled diagnostic criteria for more than one adverse outcome (*n* = 14). Baseline characteristics of patients with and without adverse outcomes are depicted in Table 1. Patients who developed an adverse maternal outcome had a lower body mass index (BMI) (23.9 vs. 26.8) and were admitted earlier than patients without an adverse outcome. Women with an adverse maternal outcome delivered about 2 weeks earlier (34 weeks (IQR 31.3–36.8) versus 36.1 weeks (34–37.5)) with a lower birth weight (1800 g (IQR 1210–2525) vs. 2300 g (1700–2975)) than those without an adverse outcome. Significant differences in laboratory parameters at admission were observed for AST (GOT) and uric acid.

### 3.1. Association of EASIX with Adverse Outcomes

Patients with adverse outcomes had higher levels of lactate dehydrogenase (282 (IQR 249–328) vs. 256 (221–305)), higher creatinine (0.7 (IQR 0.59–0.82) vs. 0.61 (0.54–0.69)), and lower platelets (181 (IQR 144–218) vs. 219 (174–258)) (Table 2). In comparison to patients without an adverse outcome, the EASIX value at admission was significantly higher in patients who subsequently developed adverse outcomes (1.1 (IQR 0.74–1.48) vs. 0.73 (0.53–1.08)) (Table 2). The median absolute change per day in lactate dehydrogenase (0 (−14–25.3) vs. −2.6 (−17.1–5)) and creatinine (0.01 (−0.001–0.03) vs. 0.002 (−0.01–0.01)) was significantly different in patients with adverse outcomes versus women without outcomes. Similarly, the absolute change per day in EASIX significantly differentiated women with adverse outcomes from those without, with median values of 0.03 (IQR −0.03–0.21) versus 0.004 (IQR −0.04–0.04) (*p* < 0.0001) (Table 2). There was no difference in the absolute change per day in levels of platelets between the two groups, suggesting that the longitudinal change in EASIX was largely driven by alterations in LDH and creatinine.

After adjustment for clinical covariates, EASIX was independently associated with adverse outcomes (aOR 2.81, 95% CI [1.86; 4.37], and *p* < 0.0001) (Supplementary Table S1). Similarly, the absolute change per day was associated with adverse outcomes (aOR 4.35, 95% CI [1.77; 12.05], and *p* = 0.003). Based on the multivariable logistic regression models, the receiver operating characteristics curves demonstrated moderate discrimination for the first EASIX with an area under the curve of 0.74 (95% CI 0.68–0.81) (*p* < 0.0001) (Table 3). The corresponding AUC for the absolute change per day in EASIX was lower (AUC 0.69, 95% CI [0.63; 0.76], and *p* < 0.0001). Combination of the first EASIX and the absolute change per day in EASIX yielded an AUC of 0.76 (95% CI [0.70; 0.82]).

Perinatal adverse outcomes were analyzed as secondary outcome and occurred in 145 patients (33%). While the absolute change in EASIX per day was significantly associated with adverse perinatal outcomes in univariable analysis (OR 2.37, 95% CI [1.10–5.49], and *p* = 0.034), this association did not persist after multivariable adjustment (Supplementary Table S2).

### 3.2. Longitudinal Dynamics of EASIX

**Figure 1:**
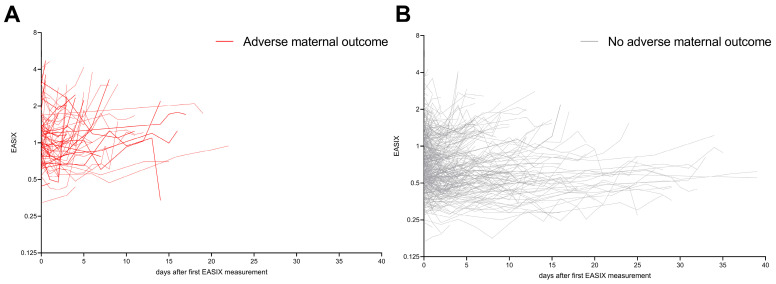
Spaghetti plots showing the individual longitudinal change in EASIX (Endothelial Activation and Stress Index) in (**A**) patients with adverse maternal outcome and (**B**) in patients without adverse outcome. The X axis shows the days after admission. Each line represents EASIX trajectories in one patient.

In total, 1733 EASIX measurements of 443 patients were included in the longitudinal analyses. The spaghetti plots illustrate individual longitudinal dynamics of EASIX stratified for the occurrence of adverse maternal outcomes (Figure 1). In the majority (66%) of patients with an adverse outcome, EASIX increased over time. The mean time from first to the last EASIX measurement was 4.6 days in patients who developed an adverse outcome and 6 days in those who did not. Our analyses included EASIX measurements until delivery or prior to an adverse outcome, whichever came first.

**Figure 2:**
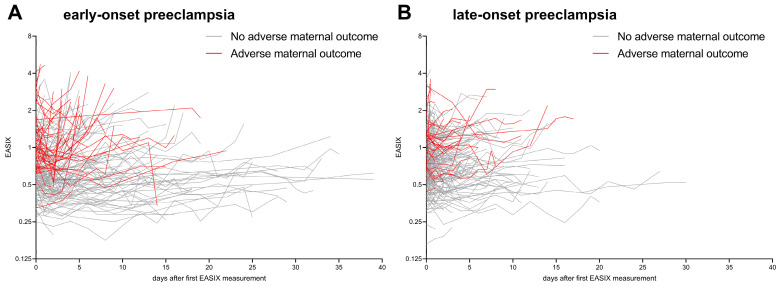
Spaghetti plots showing the individual longitudinal change in EASIX (Endothelial Activation and Stress Index) stratified for early-onset (before 34 weeks of gestation) (**A**) and late-onset preeclampsia (after 34 weeks) (**B**). Each line represents EASIX trajectories in one patient.

Figure 2 displays individual EASIX trajectories stratified for early-onset and late-onset preeclampsia based on the diagnosis before versus after 34 weeks of gestation. We observed a significantly steeper increase in EASIX among patients with early-onset preeclampsia compared with late-onset preeclampsia (0.007 (IQR −0.011–0.068) vs. 0.004 (IQR −0.053–0.042); *p* = 0.0147).

**Figure 3:**
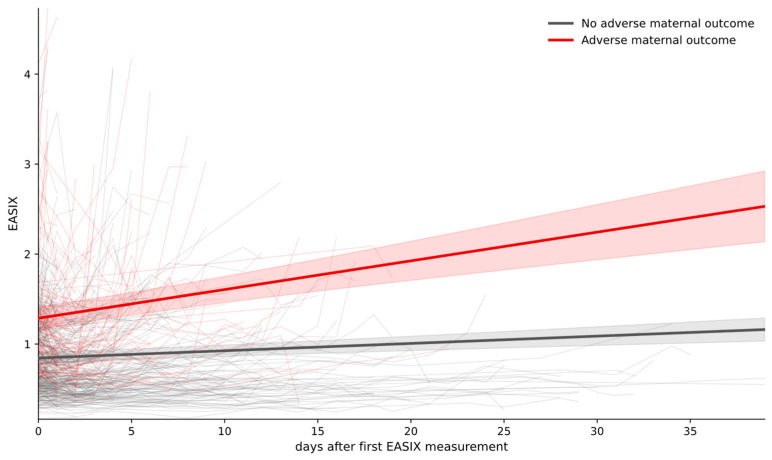
Longitudinal EASIX (Endothelial Activation and Stress Index) trajectories stratified by adverse maternal outcome. Each fine line represents an individual patient trajectory. Bold lines represent fixed-effects predictions derived from the linear mixed-effects model. Shaded areas indicate 95% confidence intervals.

Linear mixed regression modeling demonstrated that EASIX trajectories diverged over time (Table 4). Among patients without adverse outcomes, EASIX increased modestly by 0.008 units per day (β = 0.008, 95% CI [0.005; 0.011], and *p* < 0.0001). Patients with adverse maternal outcome showed significantly higher EASIX levels at admission (β = 0.443; 95% CI [0.301; 0.584]) and a steeper increase in EASIX per day (time × outcome interaction: β = 0.024/day, 95% CI [0.013, 0.034], and *p* < 0.0001). This corresponds to a 0.024 units/day faster rise in EASIX among patients with adverse outcomes compared to those without, yielding a total rate of increase of 0.032 units/day (0.008 + 0.024) in the adverse outcome group (Figure 3).

### 3.3. Sensitivity Analyses

Residuals of the primary model showed deviation from normality (Shapiro–Wilk W = 0.777); a sensitivity analysis using log-transformed EASIX yielded substantially improved residual normality (W = 0.951) (Supplementary Figure S2) and confirmed both the steeper EASIX trajectories in patients with adverse outcomes (β = 0.024, *p* < 0.001 vs. β = 0.015, *p* < 0.001) and the difference in EASIX at baseline between outcome groups (β = 0.44, *p* < 0.001 vs. β = 0.40, *p* < 0.001), supporting the robustness of the primary analysis (Supplementary Table S3).

Since increasing EASIX levels in patients with adverse outcomes may partly reflect imminent disease progression rather than independent prognostic information, we performed a sensitivity analysis excluding EASIX values obtained on the same day as the diagnosis of an adverse maternal event. This analysis yielded similar findings, with a significantly steeper increase in EASIX per day in patients who developed adverse maternal outcomes (time × outcome interaction: β = 0.011/day, 95% CI [0.001, 0.022], and *p* = 0.041), as shown in Supplementary Table S4.

Given the overlap between EASIX components and diagnostic criteria for acute kidney injury (AKI) and HELLP syndrome as part of the composite adverse maternal outcome, we added a sensitivity analysis excluding patients with AKI and/or HELLP syndrome. In this subgroup (*n* = 23 patients with adverse outcome), EASIX showed a significantly steeper increase over time compared to patients without adverse outcomes (β = 0.014; *p* = 0.048), whereas no significant difference in baseline EASIX levels was observed (β = 0.105; *p* = 0.339) (Supplementary Table S5).

In an additional sensitivity analysis, we stratified by early- versus late-onset preeclampsia. In both subgroups, baseline EASIX levels were significantly higher in patients with adverse outcomes than in those without. Interestingly, a significantly steeper EASIX trajectory in patients with adverse outcomes was observed only in the early-onset subgroup (β = 0.028, 95% CI [0.013–0.042], and *p* < 0.001) (Supplementary Table S6), whereas no significant association between EASIX trajectory and adverse outcomes was found in the late-onset subgroup (β = 0.015, 95% CI [−0.002; 0.031], and *p* = 0.082) (Supplementary Table S7).

## 4. Discussion

In this study, we evaluated the longitudinal dynamics of EASIX in patients with preeclampsia and demonstrated that EASIX not only differs at admission between patients with and without adverse outcomes but also increases significantly faster over time in patients who subsequently develop adverse outcomes. The divergence of EASIX trajectories over time suggests that longitudinal monitoring may capture individual disease dynamics that are not apparent from a single measurement.

Our data confirm findings from several studies that reported quickly changing dynamics of disease severity among patients with preeclampsia and the importance of repeated biochemical assessment [24]. A retrospective cohort study of 34 patients with preeclampsia demonstrated that angiogenic markers can quickly change with a steep slope especially in women with early-onset preeclampsia [24]. Therefore, it is reasonable to assume that longitudinal changes in laboratory markers bear the chance to be associated with preeclampsia-related adverse outcomes. Our group recently reported that high EASIX levels are associated with adverse outcomes and with the shortest remaining time to delivery in patients with preeclampsia [16]. The present findings extend this observation by demonstrating that the EASIX trajectories are independently associated with adverse outcomes primarily in patients with early-onset preeclampsia, implying that EASIX may reflect laboratory features associated with endothelial dysfunction in preeclampsia. The steeper increase in EASIX among patients with early-onset preeclampsia likely reflects the more severe disease phenotype and the correspondingly higher risk of maternal complications compared with late-onset preeclampsia.

This is in line with results by Dröge et al. showing that the delta value between the first and last measurements of the sFlt-1 (Soluble fms-Like Tyrosine Kinase 1)/PlGF (Placental Growth Factor) ratio was higher in patients with adverse outcomes than in those without [21]. However, such delta values as a single summary metric may not adequately capture the clinical value of repeated biomarker measurements as a rising biomarker value may reflect imminent disease evolution rather than serving as an early predictor of adverse outcomes. Therefore, we performed an additional linear mixed-effects model revealing that EASIX trajectories diverge over time between patients with and without adverse outcomes, a dynamic that is lost when summarizing the longitudinal course into a single metric like the delta values.

Although EASIX is a new marker in obstetrics, the longitudinal course of the individual EASIX parameters has been analyzed by Binder et al., who found high stand-alone predictive accuracies for lactate dehydrogenase, creatinine, and platelet count with AUCs of up to 73% for the prediction of adverse maternal outcomes [25]. Of note, the sFlt-1/PlGF ratio reached a similarly high AUC of 0.72 (95% CI 0.62–0.81) in these analyses [25]. In comparison, the absolute change per day in EASIX yielded a comparable AUC of 0.69 for prediction of adverse outcomes in our cohort. However, the addition of the absolute change per day in EASIX to a model already containing the first EASIX measurement did not meaningfully improve discriminatory performance (AUC 0.76 vs. 0.74), suggesting that the longitudinal change carries limited prognostic information beyond the baseline level according to the logistic regression model.

### 4.1. Clinical Implications

EASIX uniquely summarizes the systemic effects of endothelial activation and dysfunction in one simple, easily available score. Our findings suggest two complementary clinical scenarios in which longitudinal EASIX monitoring may be beneficial for clinical management of preeclampsia and early detection of individual deterioration. First, patients admitted with a moderate baseline EASIX but a steeply rising trajectory may indicate a high-risk profile that warrants intensified surveillance or delivery planning. Second, a stable or declining trajectory might be reassuring even in the context of a moderately elevated baseline EASIX. It must be emphasized that these scenarios are hypothetical based on retrospective observational data and that an independent prognostic contribution of EASIX trajectories warrants confirmation in prospective studies.

### 4.2. Strengths and Limitations

Our analyses of EASIX and its longitudinal trajectory highlight the dynamic nature of endothelial dysfunction in preeclampsia. However, given the descriptive nature of our analyses, we cannot draw conclusions for daily clinical practice and management of preeclampsia solely based on longitudinal changes in EASIX. Clinical utility of longitudinal changes remains to be questioned as the increase in a biomarker may partly reflect imminent disease progression rather than independent prognostic information. Nevertheless, our sensitivity analyses support the notion that the observed associations are not solely attributable to criterion overlap or to the proximity of the last EASIX measurement to the adverse outcome diagnosis.

The influence of ethnicity could not be evaluated adequately due to inconsistent documentation and presumably few patients with a non-Caucasian background. Although gestational age was included as a covariate in all models, residual confounding by preeclampsia phenotype cannot be excluded. Our results do not necessarily suggest that adjusting the clinical management based on the longitudinal change in EASIX would lead to improved outcomes. Given the retrospective, exploratory nature of this study and the relatively limited sample size, formal bootstrapped internal validation was not performed, possibly leading to optimism bias due to overfitting. However, this is the first study that uses serial EASIX measurements to document a worsening endothelial function in women diagnosed with preeclampsia and may stimulate further studies to confirm and validate our findings in prospective studies.

## 5. Conclusions

In patients with preeclampsia, EASIX demonstrates a dynamic longitudinal profile with significantly steeper trajectories in women who subsequently developed adverse maternal outcomes. Both the baseline level and the rate of EASIX increase are independently associated with adverse outcomes, reflecting the dynamic nature of systemic endothelial dysfunction in preeclampsia. The divergence of EASIX trajectories over time suggests that longitudinal monitoring may capture individual disease dynamics that are not apparent from a single measurement. Since EASIX is based on routine laboratory parameters, serial measurements are easily available and cost-effective, making longitudinal EASIX monitoring a promising candidate for prospective evaluation as a surveillance tool in preeclampsia management.

## Figures and Tables

**Table 1:** Baseline characteristics of patients with and without adverse maternal outcomes.

Variable	No Adverse Maternal Outcome *n* = 362		Adverse Maternal Outcome *n* = 81		*p* Value
Maternal age (years)	32	(28–36)	30	(27–35)	0.166
Gestational age at EASIX measurement (weeks)	35.1	(32.6–36.7)	33.3	(30.3–35.9)	0.0005
Pre-pregnancy body mass index (kg/m^2^)	26.8	(23–32.4)	23.9	(21.3–30.5)	0.004
Nulliparous	247	(68.2)	62	(76.5)	0.145
Use of assisted reproductive technology	34	(9.4)	8	(9.9)	0.971
Multiple gestation	33	(9.1)	11	(13.6)	0.303
Gestational diabetes	69	(19.1)	8	(9.9)	0.052
Diabetes mellitus	20	(5.5)	2	(2.5)	0.285
Chronic hypertension	47	(13.0)	14	(17.3)	0.372
History of hypertensive disorder of pregnancy	52	(14.4)	7	(8.6)	0.705
**Birth characteristics**					
Gestational age at delivery (weeks)	36.1	(34–37.5)	34	(31.3–36.8)	<0.0001
Birth weight (g)	2300	(1700–2975)	1800	(1210–2525)	<0.0001
**Laboratory parameters at admission**					
C-reactive protein (mg/L)	5.9	(3–11.1)	5.6	(2.4–11.9)	0.661
AST (GOT) (U/L)	24	(19–33)	31	(22.5–48)	<0.0001
ALT (GPT) (U/L)	14.5	(10–22)	22	(14–37)	<0.0001
Uric acid (mg/dL)	5.6	(4.8–6.3)	6	(5.4–7.3)	0.0003

Data are presented as either absolute and relative numbers or median and interquartile range (IQR) and were compared with Mann Whitney U or Chi Square test where appropriate. EASIX: Endothelial Activation and Stress Index. AST: Aspartate aminotransferase. GOT: Glutamic oxaloacetic transaminase. ALT: Alanine aminotransferase. GPT: Glutamate pyruvate transaminase.

**Table 2:** EASIX and its components stratified by adverse maternal outcome.

	No Adverse Maternal Outcome	Adverse Maternal Outcome	*p* Value
	Median (IQR)	Median (IQR)	
LDH, first (U/L)	256 (221–305)	282 (249–328)	0.0003
LDH, absolute change per day	−2.6 (−17.1–5)	0 (−14–25.3)	0.013
Creatinine, first (mg/dL)	0.61 (0.54–0.69)	0.7 (0.59–0.82)	<0.0001
Creatinine, absolute change per day	0.002 (−0.01–0.01)	0.01 (−0.001–0.03)	0.0034
Platelets, first (10^9^ cells/L)	219 (174–258)	181 (144–218)	<0.0001
Platelets, absolute change per day	−1.98 (−9.63–3)	−2.8 (−9.63–3)	0.623
EASIX, first	0.73 (0.53–1.08)	1.1 (0.74–1.48)	<0.0001
EASIX, absolute change per day	0.004 (−0.04–0.04)	0.03 (−0.03–0.21)	0.001

Mann Whitney U test was used for testing significance. IQR: interquartile range. LDH: lactate dehydrogenase. EASIX: Endothelial Activation and Stress Index. Calculation of the absolute change per day included EASIX values only before diagnosis of an adverse outcome.

**Table 3:** Multivariable receiver operating characteristics analysis for prediction of adverse maternal outcomes.

	AUC [95% CI]
EASIX, first	0.74 [0.68; 0.81]
EASIX, absolute change per day	0.69 [0.63; 0.76]
EASIX first + EASIX absolute change per day	0.76 [0.70; 0.82]

Models were adjusted for gestational age, pre-pregnancy body mass index, maternal age, and time between last and first EASIX (Endothelial Activation and Stress Index) measurement. AUC: area under the curve. CI: confidence interval.

**Table 4:** Linear mixed-effects model assessing factors influencing EASIX (Endothelial Activation and Stress Index) over time.

	β Estimate	±SE	95% CI	*p* Value
Intercept	0.501	±0.323	−0.131; 1.133	0.120
Age	0.009	±0.005	−0.000; 0.019	0.051
Body Mass Index	−0.01	±0.004	−0.017; −0.002	0.011
Gestational age at admission	0.01	±0.007	−0.004; 0.024	0.175
Time	0.008	±0.002	0.005; 0.011	<0.0001
Outcome	0.443	±0.072	0.301; 0.584	<0.0001
Time x Outcome	0.024	±0.005	0.013; 0.034	<0.0001

SE: standard error. CI: confidence interval. The β estimate for “Time” indicates the time since first EASIX measurement and thus the increase in EASIX in patients without adverse outcomes. The β estimate for “Outcome” indicates the difference in EASIX at admission between patients with vs. without outcomes. The β estimate for “Time × Outcome” indicates the difference between EASIX changes over time between patients with vs. without outcomes. Age, BMI (body mass index), and gestational age at admission were included as covariates.

## Data Availability

The data presented in this study are available from the corresponding author upon reasonable request due to ethical reasons.

## Supplementary Materials

The following supporting information can be downloaded at https://www.mdpi.com/article/10.3390/diagnostics16132007/s1: Figure S1: Patient flow chart; Figure S2: Residual diagnostics of the linear mixed-effects model for EASIX; Table S1: Univariable and multivariable models for prediction of adverse maternal outcome; Table S2: Univariable and multivariable models for prediction of adverse perinatal outcome; Table S3: Sensitivity analysis of linear mixed-effects model using log-transformed EASIX; Table S4: Sensitivity analysis of linear mixed-effects model after excluding EASIX values obtained on same day as diagnosis of maternal adverse outcome; Table S5: Sensitivity analysis of linear mixed-effects model after excluding patients with acute kidney injury and/or HELLP syndrome to exclude overlap with EASIX components; Table S6: Sensitivity analysis of linear mixed-effects model in patients with early-onset preeclampsia; Table S7: Sensitivity analysis of linear mixed-effects model in patients with late-onset preeclampsia.

## Abbreviations

The following abbreviations are used in this manuscript:

EASIX	Endothelial Activation and Stress Index
HELLP	Hemolysis, Elevated Liver enzymes, Low Platelets (HELLP) syndrome
LDH	Lactate Dehydrogenase
sFlt-1/PlGF ratio	The sFlt-1 (Soluble fms-Like Tyrosine Kinase 1)/PlGF (Placental Growth Factor) ratio
BMI	Body Mass Index
