# Supplementary material for: Longitudinal Changes in the Endothelial Activation and Stress Index (EASIX) in Patients with Preeclampsia
Source: Diagnostics (Basel). 2026 Jun 27;16(13):2007. doi: 10.3390/diagnostics16132007 (PMC13359994; doi:10.3390/diagnostics16132007)
Supplement: Supplementary file 1 [file diagnostics-16-02007-s001.zip › diagnostics-4322714-supplementary.pdf]

Figure S1 Patient flow chart

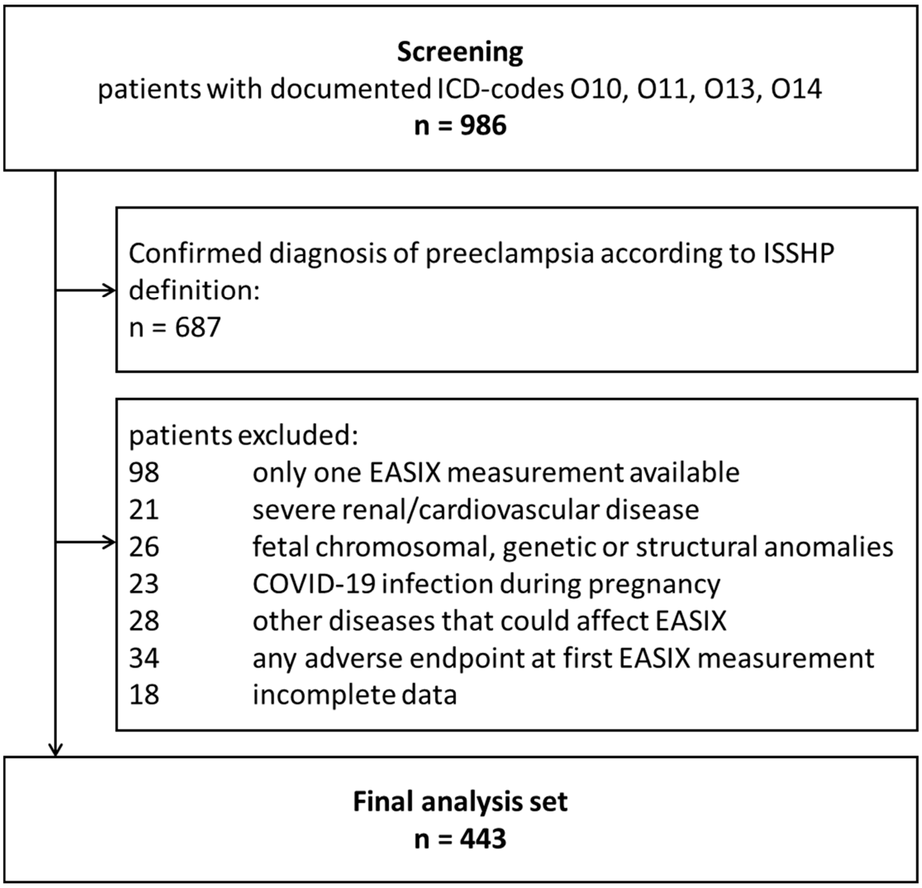

ISSHP: International Society for the Study of Hypertension in Pregnancy. EASIX: Endothelial Activation and Stress Index. ICD: International Classification of Diseases.

Figure S2 Residual diagnostics of the linear mixed-effects model for EASIX (Endothelial Activation and Stress Index). (A) Quantile-quantile plot (left) and residuals vs fitted values (right) fitted on untransformed EASIX (Shapiro Wilk W=0.777). (B) Corresponding diagnostic plots for the model fitted on log-transformed EASIX, indicating better model assumptions compared to the untransformed model (Shapiro Wilk W=0.951).

A

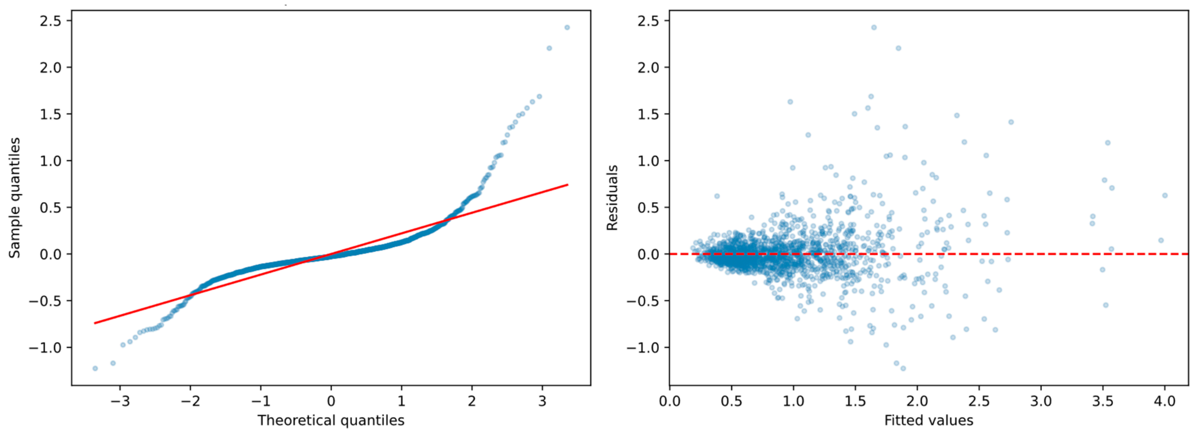

B

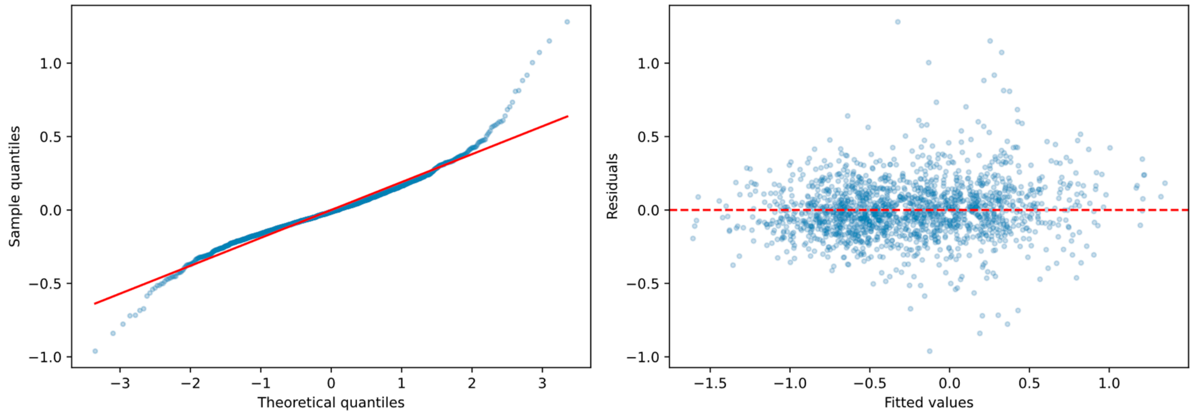

Table S1 Univariable and multivariable models for prediction of adverse maternal outcome.

	OR	95% CI	p value	aOR*	95% CI	p value
EASIX, first	2.88	(1.97 – 4.32)	<0.0001	2.81	(1.86 – 4.37)	<0.0001
EASIX, absolute change per day	6.25	(2.49 – 17.36)	0.0002	4.35	(1.77 – 12.05)	0.003

* adjusted for gestational age, pre-pregnancy body mass index, maternal age, and time between last and first EASIX measurement. EASIX: Endothelial Activation and Stress Index. OR: Odds Ratio. aOR: adjusted Odds Ratio. CI: confidence interval.

Table S2 Univariable and multivariable models for prediction of adverse perinatal outcome.

	OR	95% CI	p value	aOR*	95% CI	p value
EASIX, first	1.39	(0.996 – 1.95)	0.052	1.02	(0.55 – 1.92)	0.95
EASIX, absolute change per day	2.37	(1.1 – 5.49)	0.034	1.53	(0.47 – 4.55)	0.45

* adjusted for gestational age, pre-pregnancy body mass index, maternal age, and time between last and first EASIX measurement. EASIX: Endothelial Activation and Stress Index. OR: Odds Ratio. aOR: adjusted Odds Ratio. CI: confidence interval.

Table S3 Sensitivity analysis of linear mixed-effects model using log-transformed EASIX.

	**β estimate**	**± SE**	**95% CI**	**p value**
Intercept	-0.738	0.288	-1.302; -0.174	0.01
Age	0.011	0.004	0.002; 0.019	0.014
Body Mass Index	-0.012	0.003	-0.019; -0.005	<0.0001
Gestational age at admission	0.012	0.006	-0.000; 0.025	0.051
Time	0.011	0.001	0.008; 0.013	<0.001
Outcome	0.405	0.064	0.279; 0.531	<0.001
Time x Outcome	0.015	0.004	0.006; 0.023	<0.001

SE: standard error. CI: confidence interval. EASIX: Endothelial Activation and Stress Index. Age, body mass index, and gestational age at admission were included as covariates.

Table S4 Sensitivity analysis of linear mixed-effects model after excluding EASIX values obtained on same day as diagnosis of maternal adverse outcome.

	**β estimate**	**± SE**	**95% CI**	**p value**
Intercept	0.477	0.323	-0.157; 1.11	0.14
Age	0.01	0.005	0.0004; 0.019	0.042
Body Mass Index	-0.01	0.004	-0.017; -0.002	0.012
Gestational age at admission	0.01	0.007	-0.004;0.024	0.163
Time	0.008	0.002	0.005; 0.011	<0.001
Outcome	0.445	0.073	0.303; 0.587	<0.001
Time x Outcome	0.011	0.006	0.001; 0.022	0.041

SE: standard error. CI: confidence interval. EASIX: Endothelial Activation and Stress Index. Age, body mass index, and gestational age at admission were included as covariates.

Table S5 Sensitivity analysis of linear mixed-effects model after excluding patients with acute kidney injury and/or HELLP syndrome to exclude overlap with EASIX components.

	**β estimate**	**± SE**	**95% CI**	**p value**
Intercept	0.443	0.31	-0.165; 1.05	0.154
Age	0.012	0.005	0.003; 0.02	0.01
Body Mass Index	-0.008	0.004	-0.016; -0.001	0.019
Gestational age at admission	0.008	0.007	-0.005; 0.021	0.244
Time	0.008	0.001	0.006; 0.011	<0.001
Outcome	0.105	0.11	-0.11; 0.32	0.339
Time x Outcome	0.014	0.007	0.0001; 0.028	0.048

SE: standard error. CI: confidence interval. EASIX: Endothelial Activation and Stress Index. Age, body mass index, and gestational age at admission were included as covariates. The group of adverse maternal outcomes included 23 patients.

Table S6 Sensitivity analysis of linear mixed-effects model in patients with early-onset preeclampsia.

	**β estimate**	**± SE**	**95% CI**	**p value**
Intercept	0.222	0.599	-0.942; 1.395	0.711
Age	0.0001	0.009	-0.017; 0.017	0.992
Body Mass Index	-0.007	0.007	-0.02; 0.007	0.346
Gestational age at admission	0.026	0.016	-0.005; 0.056	0.098
Time	0.009	0.002	0.004; 0.013	<0.001
Outcome	0.508	0.114	0.284; 0.732	<0.001
Time x Outcome	0.028	0.007	0.013; 0.042	<0.001

SE: standard error. CI: confidence interval. Age, body mass index, and gestational age at admission were included as covariates.

Table S7 Sensitivity analysis of linear mixed-effects model in patients with late-onset preeclampsia.

	**β estimate**	**± SE**	**95% CI**	**p value**
Intercept	0.263	0.719	-1.147; 1.672	0.715
Age	0.016	0.006	0.005; 0.027	0.004
Body Mass Index	-0.012	0.004	-0.02; -0.003	0.009
Gestational age at admission	0.012	0.018	-0.024; 0.047	0.522
Time	0.007	0.003	0.001; 0.012	0.014
Outcome	0.379	0.092	0.198; 0.561	<0.001
Time x Outcome	0.015	0.008	-0.002; 0.031	0.082

SE: standard error. CI: confidence interval. Age, body mass index, and gestational age at admission were included as covariates.
